# Behavioral Skills Training in Portuguese Children With School Failure Problems

**DOI:** 10.3389/fpsyg.2018.00437

**Published:** 2018-05-29

**Authors:** Edgar Galindo, Adelinda A. Candeias, Heldemerina S. Pires, Luísa Grácio, Marcus Stück

**Affiliations:** ^1^Centro de Investigação em Educação e Psicologia, Departamento de Psicologia, Universidade de Evora, Evora, Portugal; ^2^Department of Childhood Pedagogy, DPFA Hochschule Sachsen, University of Applied Sciences, Leipzig, Germany

**Keywords:** school failure, academic behavior, applied behavior analysis, behavioral skills training, educational psychology

## Abstract

This paper postulates that psychology can make an important contribution at an individual level to help children with school failure problems in a context where too little applied research has been conducted on the instructional needs of these children. Some data are analyzed, revealing that, despite some progress, school failure is still a main educational problem in many countries. In this study, Behavioral Skills Training (BST) was applied in Portugal to train children with school failure difficulties. BST is a method based on Applied Behavior Analysis, a teaching package consisting of a combination of behavioral techniques: instructions, modeling, rehearsal, and feedback. Two empirical studies are presented. Their main purpose was to develop behavioral diagnostic and training techniques to teach lacking skills. School success was defined in terms of a set of skills proposed by teachers and school failure as a lack of one or more of these skills. The main instrument was a package of training programs to be applied in three areas: basic behavior (precurrents), academic behavior, or social behavior. The second instrument is a package of check-lists, aimed to determine the level of performance of the child in an area. This check-list was applied before (pre-test) and after (post-test) training. In the first study, 16, 7- to 8-year old children were trained. They were attending the second or third grades and having academic difficulties of different origins. The effects of the training programs are evaluated in terms of percentage of attained objectives, comparing a pre- and a post-test. The results showed an increase in correct responses after training in all cases. To provide a sounder demonstration of the efficacy of the training programs, a second study was carried out using a quasi-experimental design. A multiple baseline design was applied to three 10- to 11-year-old children, referred by teachers because of learning difficulties in the fourth grade. Results showed few performance changes without training. Increases in behavior following BST were evident in all cases, indicating that training generated improvement in all three children. In both studies, comparable results occurred across students, demonstrating replication of the effects of the training programs.

## Introduction

There are several definitions of school failure. Fernandez Enguita et al. (2010, p. 18) propose one, which is adequate to our purposes:

…*school failure* is the situation of the student who, in trying to achieve the minimum objectives set forth by the institution, (…) fails to do so and withdraws after having been categorized as a failure…

Nevertheless, in this article a pragmatic working definition has been chosen: children with school failure are those referred by the teachers because they have repeated grades at least once or because they are currently at risk of having to repeat a grade.

School failure is a main educational problem in Portugal. Recent data by the European Commission/Directorate-General for Education and Culture (EC DGEC) (2016, p. 39) show that Portugal has one of the highest rates of early school leaving (as measured by the number of individuals 18–24 years of age that did not finish school and do not attend school for each 100 individuals of the same age). The Portuguese figure of 14% compares to 3% in Croatia and 11% percent across the UE. In spite of improvements since 2005, early school leaving is still a main problem in Portugal. Fourteen 14% of people between 18 and 24 years who have not finished have, nevertheless, quit attending.

These data do not address the reasons behind the phenomenon. Yet, it might be supposed that at least 14% of children had some kind of academic problem at an early school age. Dropping out of school is largely determined by school failure in primary school (Justino et al., 2014, p. 54).

There are several types of school failure in Portugal: early school leaving, successive failure leading to a gap between grade and the chronological age of children (rate of retention), and dropping out of primary schooling (10–15 years). Table 1 compares some aspects of the problem in Portugal and other European countries for 2009. At the primary school level (ISCED 1) Portugal had by far the greatest proportion of pupils repeating at least once year of primary education, (22.4%), vs. 12.2% for the next highest, Spain, and 7.7% for the European mean. This means that, in Portugal, 22.4% of children between 6 and 12 years have some kind of learning problems at school.

**Table 1:** Proportion of 15 years old pupils who had repeated at least once in primary education (ISCED level 1) in 2009, a selection of some countries (Adapted from Eurydice, 2011, p. 36).

**EU-27**	**BG**	**CZ**	**DK**	**DE**	**FR**	**ES**	**IT**	**AT**	**PT**
7.7	2.7	2.1	3.6	9.2	17.8	12.2	1.0	4.9	22.4

*EU, European Union; BG, Bulgaria; CZ, Czech Republic; DK, Denmark; DE, Germany; FR, France; ES, Spain; It, Italy; AT, Austria; PT, Portugal*.

Additional data show an urgent need for intervention in the early school years, such as Portuguese sources (Gabinete de Estatística e Planeamento da Educação, 2010, p. 18) that point out that the yearly rate of retention and dropouts at basic school in 2008 was 7.6 for ISCED 1 (grades one to four) in 2008. These data show significant improvement from 1999 to 2008, as retention and dropout rates fell from 12.1 to 7.6%. Nevertheless, the rate remains significantly high, since it means that, in the first three grades, 7.6% of children either drop out of school or do not pass to the next school year.

The Organization for Economic Cooperation and Development [OECD] (see OECD, 2010), the European Union and international educational agencies identified the problem many years ago. A set of causes and recommendations for fighting school failure were proposed.

Commonly cited causes included cognitive deficiencies of children, unstable family environments (conflicts, divorce, parental disorders), socioeconomic issues such as low SES (poverty or social exclusion), and then, inadequate quality or functioning of the educational system (bad teaching methods, faulty school organization, or unorganized curricula; Webster-Stratton et al., 2008). The failure of any one child is most likely the result not of one factor but rather the interaction of many, as in the case of the children participating in this study. However, from the point of view of treatment, the cause of school failure is not especially pertinent. It is urgent to find intervention strategies able to cope with the problem independently of the cause.

Scientists have proposed several strategies to deal with the problem, such as increased preschool education, adapting schools to children's needs and possibilities, preparing more and better teachers and specialists, innovating teaching and evaluation methods, improving relations between school and the social environment or between schools and families (Carbonero et al., 2017), and applying personalized teaching methods, according to specific needs and characteristics of each child (Marchesi and Hernández Gil, 2003). This last proposal is the basis of our work. At an individual level, the one main task of psychology is to develop differential teaching methods to be applied as early as possible. On the other hand, very little applied research has been conducted on the instructional needs of these children. Poche et al. (1981, p. 323) observed that “Students with learning disabilities who experience difficulties in mathematics are frequently taught multiplication facts using the same procedures and sequences that are used with students without difficulties, that is, repetition drills…” This also appears to be true for children with problems of school failure, who are frequently taught reading, writing and arithmetic with the same methodology that has failed to work in the past.

### Applied behavior analysis and behavioral skills training

Applied Behavior Analysis (ABA), also called Behavior Modification, is the applied part of the Experimental Analysis of Behavior, based on the principles of conditioning as they were defined originally by I. P. Pavlov and B. F. Skinner. ABA techniques have proved to be useful tools for coping with a broad range of behavior problems. A great deal of research is available about the application of ABA to the training and rehabilitation of people with different kinds of learning difficulties. Authors like Whitman et al. (1971) came up with this process while training mentally retarded children in instructions following, imitation, selfcare, eating, verbal, and social behavior. Since then, behavioral programs have been applied successfully to train people with intellectual disabilities in academic skills such as reading (Saunders et al., 2003) and using computers (Jerome et al., 2007). Another example has been training people with hearing and visual impairments (Green et al., 2005; Toussaint and Tiger, 2010), and others with autism (Axe and Sainato, 2010; Rodriguez-Medina et al., 2016) or with learning disabilities (Wood et al., 1998; Levingston et al., 2009). The analysis of school failure has accompanied this development. Adelman and Taylor (1993) analyzed the problems of learning at school, including the types, causes and consequences of these problems as well as some proposed solutions, emphasizing the use of behavioral techniques. Hallahan et al. (1999) and Wallace et al. (1992) analyzed the causes of school failure, pointing to individual, family and school system factors, in much the same way. This work also identified a very important cause of individual school failure, namely, the absence of antecedent behaviors, such as language, social and cognitive repertoires, which are a prerequisite for the learning of reading, writing, and arithmetic. Research in other countries has confirmed the importance of those cognitive, behavioral, verbal, or social pre-academic competencies for the learning of reading, writing, and arithmetic in the early school years (Carroll et al., 2003; Neef et al., 2003; DiLalla et al., 2004; Leppänen et al., 2004; Connor et al., 2005; Guevara and Macotela, 2006).

Behavior Skills Training (BST) is based on Applied Behavior Analysis, which can be used to teach a wide variety of important skills to individuals of different ages and difficulties. Actually, it is a teaching package bringing behavioral techniques together which have been used for many years to train disadvantaged or disabled children, school children or adults. It is “an effective training package that consists of instructions, modeling, rehearsal, and feedback” (Ward-Horner and Sturmey, 2012, p. 75). Instructions consist of explaining how to carry out a determined task. During modeling, the task is demonstrated by the trainer while the trainee observes. Next, the trainee practices the task. A good chain of responses is reinforced, and errors are corrected in the last, or feedback, phase.

As mentioned above, the components of BST have been in used independently or together by behavior analysts since the 1970s. Nevertheless, the term was first used by Sarokoff and Sturmey (2004).

In recent decades, several researchers have used the four components of BST without using the term. Danish et al. (1976) outlined components of a “skills training” in a course of helping relationships, which were: (1) identifying behavior objectives; (2) practicing skills; (3) group discussion; (4) understanding the reason for using the skills; (5) presenting the skills; (6) active participation by trainees; (7) modeling of techniques; and (8) immediate feedback. Evidently, these eight steps are quite similar to BST. At that time, the authors described the existence of “skills training groups” that focused on teaching skills required to implement specific behaviors. In a program for parents, Gordon and Davidson (1981) applied a “behavior skills training” containing the elements of: (1) training parents to focus on observable behavior; (2) teaching parents concepts of learning theory; and (3) helping parents to apply the concepts when working with their children. Although the “behavior skills training” in these papers differs slightly from that of the BTS in ABA research, it is an important antecedent to the current behavior principles.

Other training packages contain all of the components of BST without labeling it as such. Bornstein et al. (1977) used all of the components of BST to train four non-assertive children in skills such as eye contact, loudness of speech, speech duration, and requests for new behavior. Children learned all behaviors with good results in generalization and maintenance. Minkin et al. (1976) also used the components of BST to teach a social skill, showing the effectiveness of instructions, modeling, rehearsal, and feedback to increase assertive conversational skills and general social conversation skills. Wurtele et al. (1989) used the components of BST to teach sexual abuse prevention skills to children.

Some studies have analyzed the effects of the individual components of BST, and have demonstrated the importance all four steps. In this line, Yeaton and Bailey (1983) applied a package to train guards about how to teach children to cross the street; they then analyzed the effectiveness of the different steps. They found that the rehearsal, feedback and praise components were necessary to produce effective behavioral change, while isolated components were not so effective. In a study of the prevention of child molestation, Poche et al. (1981) used modeling, rehearsal, and social reinforcement to teach children how to correct verbal and motor responses. The results revealed the success of modeling and rehearsal, when used together. Parsons and Reid (1995) used instructions, role-plays, and feedback to train supervisors. They found that staff skill performance endured as a result of feedback.

Since then, several researchers have successfully used BST to teach children a variety of skills. Johnson et al. (2005) examined successfully the effectiveness of individual behavioral skills training in teaching 13 preschool children abduction prevention skills in naturalistic settings. All the children maintained the skills in the 2-week and 1-month follow-ups. Similar results were obtained by Miltenberger et al. (2009) and Pan-Skadden et al. (2009). Homlitas et al. (2014) evaluated the effectiveness of a behavioral skills training package to teach the picture exchange communication system (PECS) to teachers employed at a therapeutic center for children with autism. Similar results were obtained by Barnes et al. (2011), Gianoumis et al. (2012), and Nigro-Bruzzi and Sturmey (2010) with children with autism. Seiverling et al. (2012) used successfully a package of BST to teach parents of 3 children with autism to apply a treatment for food selectivity. Results were reflected in increases in children food acceptance and decreases in disruptive behavior. Similar results were obtained by Mueller et al. (2003). Miles and Wilder (2009) applied BST to increase correct implementation of guided compliance by caregivers of 3 children with autism who exhibited noncompliance. The skills were successfully trained and they were exhibited in different settings 3–6 weeks after training ended.

Galindo et al. (2013) and Galindo (2009) applied ABA techniques to train children with intellectual, sensorial, social, and academic deficiencies. These works are the direct antecedents of the current project. The current study extends BST research to students experiencing school failure, adapts the procedures to children at educational risk, and evaluates the intervention with a multiple baseline.

### The present study

The current research on school failure was carried out in two different phases. The first interventions were carried out in low income, public elementary schools surrounding Lisbon. Participating children were often victims of social exclusion, i.e., members of ethnic minorities or living in extreme poverty. In one case, the parents were often absent. Data from this period have been published (Galindo, 2015). The second phase began in 2013 and applied the same package of Behavioral Skills Training to another type of child, residents of the Alentejo, a disadvantaged region of Portugal. Results of this second intervention are analyzed below. Nevertheless, a global explanation of the intervention strategies in both phases will be given, in order to provide a clearer image of the field.

The main goal of these studies was to develop behavioral diagnostic and training techniques to teach academic and social skills to children with school failure problems. School success was defined as a set of skills proposed by teachers. School failure was then understood as a lack of one or more of these skills. The skills to be mastered by a single child were defined in terms of behavioral objectives, according to the expectations of the teacher. The nature, scope and probable origins of the child's academic problems were extremely varied.

The specific goals of these interventions were:

1. To develop behavioral diagnostic techniques based on school defined skills for elementary school (ISCED 1) in Portugal.
2. To develop skill-based training programs.

On the basis of a precise description of the problems of each child, one (or more) intervention program(s) was specifically tailored for that learner, following the logic of Behavioral Skills Training. A token economy was introduced to motivate children (Ayllon and Azrin, 1968), i.e., children earned points through good responses. The points were then exchanged for privileges chosen by the children (for instance, playing with the teacher).

Three main areas of intervention were defined on the basis of preliminary observations of academic problems at school:

1. Basic behavior. Pre-current behaviors, i.e., social and pre-academic skills that constitute the basis for learning other skills.
2. Academic behavior. Portuguese reading and writing, arithmetic, and the environment (social studies and science).
3. Social behavior. Social skills for proper school functioning.

Children referred to the program by teachers for having school failure problems were evaluated and trained on an individual basis by specially trained psychology or special education students (tutors). These tutors mentored one child during a semester (15–16 weeks) and worked with them for 2–4 h a week at school. All skills training programs had the same structure (see Table 2), including a pre and a post-test. Consequently, results were evaluated mainly by comparing the percentage of skills which were defined in terms of attained objectives in pre- and post-tests. However, training time was also taken into account.

**Table 2:** Program to recognize letters.

General Objective
The child must be able to recognize all letters of the Portuguese alphabet
Specific objectives
The child must be able to identify and reproduce the sound of all alphabet letters
handwritten, lower case letter
handwritten, capital letters
print, lower case letters and print, capital letters
Definition
A response is correct if the child
identifies a letter presented by the tutor
reproduces the sound of a letter presented by the tutor
Precurrents
Basic repertoires and language without articulation problems
Materials
Cards with letters (see Table 3)
Place
Working room or classroom
Procedure
Pre-test: All letters are presented
Training: A package of instructions, modeling, rehearsal, and feedback
handwritten, lower case letters
handwritten, capital letters
print, lower case letters
print, capital letters
The child must be able to respond correctly, without help, to the same question in five successive trials
Post-test: All letters are presented
Training has been successful if the child attains at least 80% of correct responses


The training programs were highly varied, since the nature of target skills was widely different. For example, “reading” means recognizing letters for one child, but spelling words for another one. This means leads to wide variation in the reading intervention for each child under the same label. Additionally, some children received training in one subject, some in two, some in three, and so on. Training duration also varied. The data comparing pre and post-test measures showed a positive effect in most cases but, evidently, a sounder demonstration of the efficacy of the program was necessary.

Consequently, a study was carried out with a quasi-experimental, multiple baseline design in order to reinforce the scientific bases of the skills training programs. Data from this study suggested that the progress by participant children was the result of the intervention programs rather than other factors (Galindo, 2015). A replication of this study will be presented below.

## Training children with school failure problems in the alentejo: study 1

An average of 14 children was trained every semester between 2013 and 2016. In the following description, only data from one semester (2013–2014) is analyzed.

### Materials and methods

#### Participants and setting

Sixteen children aged seven to eight in the second and third grades were referred to the program due to academic problems (see Table 4). All of them were at risk of failing the school year. They had similar SES, as they belonged to the lower middle class of a small Portuguese town. The causes of their problems were difficult to determine. Nevertheless, according to their teachers, family problems (like divorce or separation of the parents) were often present. The training took place at a public elementary school in Evora, a small city in the Alentejo region. Sessions were conducted at tables located in school corners.

#### Instruments

The main instrument is a package of behaviorally designed training programs to be applied in three areas, namely, basic behavior (precurrent), academic behavior (subjects of Portuguese ISCED 1), or social behavior (see Tables 2, 3). Every program was designed to solve a specific problem (writing, spelling, articulation, etc.). Tutors are explicitly instructed to develop their own programs on the basis of the pre-existing musters (Galindo, 2015). Nevertheless, all programs had the same structure: (1) a general objective, (2) skills defined in terms of specific objectives (a set of correct responses to be given by the child), (3) a definition of the previous skills necessary to learn the new skill (precurrents), (4) a pre-test (% of attained objectives/correct responses), (5) a training package based on instructions, modeling, rehearsal, and feedback, (6) a post-test (% of attained objectives/correct responses). The specific objectives of the program are defined in terms of responses given by a specific child. Every program has a set of previously established correct responses Evaluation of the efficacy of the program was carried out by comparing the percentage of attained objectives before (pre-test) and after training (post-test).

**Table 3:** Check list: recognizing letters.

Objective
The child must identify the letters presented by the tutor
Place
Workspace or classroom
Material
Cards with letters
cards with all handwritten, lower case letters
cards with all handwritten, capital letters
cards with all printed, lower case letters
cards with all printed, capital letters
Procedure
The tutor says “we are going to play cards; I will show you four cards with letters and you must put your finger on the letter I am looking for”
Cards are chosen randomly
All letters must be shown
The child must identify the letter in <5 s
A training is necessary if the child obtains less than 80% of correct responses. In such a case, a more precise evaluation of the errors is necessary


The second instrument is a package of behaviorally designed check-lists (Galindo, 2015) intended to determine the level of performance of the child in an area, i.e., basic behavior, academic behavior, and social behavior. Every list was designed by the tutor to evaluate a specific skill of the child (e.g., their articulation problems). Nevertheless, every check-list had the same structure (see Table 3).

#### Procedure

Before any contact with children, written informed consent was obtained from the parents. Before the assessment period, children were informed of the nature of the intervention and were told that the programs were expected to help them in their school subjects. Behavioral Skills Training was applied by ABA trained Psychology students (tutors) for 2 h a week (2 sessions × 1 h) during a semester (15 weeks). Each tutor took on a child and worked with them throughout the intervention. Before the training began, the tutors gathered all the information they could about their child. Existing and non-existing skills, as well as the possible aims of the intervention were defined together with the teacher. The first week served for the tutors to get acquainted with the child, and the tutor then applied a set of check-lists to identify as precisely as possible the specific problems of the child in each subject. All information was used to elaborate a hierarchy of problems. On this basis, an intervention strategy was designed, elaborating specific training programs for the child. The specific objectives of the program were the goals of the intervention. The percentage of correct responses in a given program attained before and after intervention are the main data. The child must attain at least 80% of correct responses in a given program. During intervention, the tutor would collect the children from the classroom and work with them in another room for an hour, always in coordination with the teacher. An intervention might last a few weeks or the whole semester, depending on the children's needs. A post-test was carried out following the training.

#### Results

Table 4 shows a list of participants, the pre-test and post-test results and the number of sessions (including testing). Ten children (three girls and seven boys) were 7 years old and were in second grade. Five students (two girls and four boys) were eight and attended the third school year. They were trained mainly in school subjects like reading, writing, arithmetic, and environment, but some of them received additional training in precurrent behaviors like language (articulation), fine motor skills and good classroom social behavior (sitting in the chair and/or concentration). The objective of sitting in the chair for 30 min was defined by the teacher as good enough to work in the classroom.

**Table 4:** Study 1. Children at ISCED 1 trained in the period 2013–2014.

**Child**	**Age range**	**School year**	**Training program**	**Pre-test (% correct responses)**	**Post-test (% correct responses)**	**Sessions**
1.	7–8	2nd	Writing	55	75	18
2.	7–8	2nd	Sitting 30′	7	100	13
3.	7–8	2nd	Arithmetic	34	73	12
4.	7–8	2nd	Articulation	40	100	10
			Reading	74	100	10
5.	7–8	2nd	Reading	30	88	10
6.	7–8	2nd	Articulation	0	100	14
7.	7–8	2nd	Sitting 30′	80	100	17
8.	7–8	2nd	Environment	73	86	12
9.	7–8	2nd	Arithmetic	75	100	10
10.	7–8	2nd	Concentration in classroom	40	100	12
11.	7–8	2nd	Arithmetic	68	81	12
12.	7–8	3rd	Sitting 30′	60	85	8
13.	7–8	3rd	Fine motor skills (Pre-writing)	92	100	20
14.	7–8	3rd	Writing	43	86	13
			Arithmetic	35	100	9
15.	7–8	3rd	Articulation	66	96	12
16.	7–8	3rd	Writing	55	84	9
			Reading	33	60	9

*The table shows training programs applied to every child, the results obtained before (pre-test) and after training (post-test), as well as the number of sessions. 1 session = 1 h*.

Children 4, 14, and 16 were trained in two programs, the others in only one. Most of the children attained more than 80% of correct responses by the end of the training. Exceptions were children 1, 3, and 16, who were not able to complete the program before the end of the semester. Child 1 was trained in writing. She improved from 55 to 75% correct responses in 18 sessions (18 h), even though failing to achieve the target 80%. Child 2 was referred because of hyperactivity in the classroom. He was trained to sit still for 30 min while concentrating on a task and he advanced from 7% (2.1 min) to 100% (30 min) in 13 sessions (13 h). Child 3 received math training. He advanced from 34 to 73% in 12 sessions (12 h). Child 4 was referred because of reading problems. Our evaluation showed a language problem. Consequently, he was trained first in articulation, where he advanced from 40 to 100% in 10 sessions (10 h) and then in reading, where he advanced from 74 to 100% in 10 sessions (10 h). Child 5 was trained in reading and he advanced from 30 to 100% of correct responses in 10 sessions (10 h). Child 6 also received reading training. A detailed assessment revealed language problems and he was trained in articulation, whereupon he advanced from 0 to 100% correct responses in 14 sessions (14 h). Reading training was planned for the following semester. Child 7 was referred because of hyperactivity. Detailed observations revealed he could sit 24 min (80%). Nevertheless, he was trained in sitting and concentration, and managed to attain 100% (30 min) in 17 sessions. Child 8 was trained in environment (Natural Sciences) and she improved from 73 to 86% correct responses in 12 sessions. Child 9 was trained in math and increased from 75 to 100% in 10 sessions (10 h).

Child 10 was referred because of “lack of attention and disruptive behavior” in the classroom. Our observations revealed he could work without distraction 12 min (40%). Consequently, he was trained to concentrate on a task without getting distracted during 30 min, advancing to 100% (30 min) in 12 sessions (12 h). Child 11 received math training; he improved from 68 to 81% correct responses in 12 sessions (12 h). Child 12 was referred because of hyperactivity. Our observations showed he could sit on his chair for 18 min (60%). After 8 sessions, he was able to sit and concentrate on his work for almost 26 min (85%). Child 13 was already in the 3rd grade, but she could not hold her pen properly and had problems completing writing tasks. Our observations revealed she had few motor skills problems, but they were enough to slow her writing. She received fine motor skills training consisting of pre-writing exercises and advanced from 92 to 100% of correct responses in 20 sessions (20 h). Child 14 had problems with writing and math. It was decided to prioritize math and he received 13 training sessions (13 h) and improved from 43 to 86% of correct answers. He also received math training, where he advanced from 35 to 100% correct responses in 9 sessions (9 h). Child 15 was referred due to reading difficulties. Further analysis revealed an articulation problem and he received 12 training sessions, advancing from 66 to 96%. Child 16 had problems with reading and writing. The tutor decided to start with writing and the child advanced from 55 to 84% in 9 sessions. It was considered a good result and a training program in reading was applied, where he advanced from 33 to 60% in 9 sessions.

A comparison of pre- and post-test results showed an increase in correct responses following training in all cases. The number of sessions necessary to train a skill is highly variable, ranging from 8 to 20 sessions. For instance, articulation problems were solved in 10 sessions (child 4), in 14 sessions (child 6), or in 9 sessions (child 16).

An independent-samples *t-*test was run. It showed there are no outliers in the data, i.e., the results of children's performances were normally distributed, as assessed by Shapiro–Wilks test (*p* > 0.05). Additionally, a paired-sample *t*-test was run to determine if there were significant differences in the performance of children before training (pre-test) and after training (post-test). Table 5 shows that there was an increase in the mean values (39.31 points more) and a greater homogeneity in post-test results (less dispersion in Student deviation). A statistically significant difference in mean values was also found (*t* = −5.72, *p* > 0.000). Therefore, it is possible to say the increase in the performance of children was with 99% probability a result of the training programs.

**Table 5:** Study 1. A comparison of the performance of children before training (pre-test) and after training (post-test).

	**N**	**Mean**	**Student deviation**	**Difference mean**	**T**	**df**	**Sig. (2-tailed)**
Pre-test	16	51.2500	25.57212	39.31	25.57212	25.57212	25.57212
Post-test	16	90.5625	10.65813				

#### Discussion

The data show the positive and negative aspects of the training programs. On one hand, it seems that all training programs (independently of the problem treated) led to increased correct responses in all children (independently of gender or age), in a relatively short time (8–20 h of training). In other words, every child managed to quickly learn a previously lacking skill. It seems then that the strategy used to identify the lacking skills and to train the identified skills produced an overall positive change in children's behavior. Teachers' subjective opinions confirmed this supposition. Nevertheless, in at least two cases, the teacher complained the children were “behaving better” but lacked any obvious improvements in academic performance. This is probably due to the fact that teachers were more focused on concrete results in academic subjects and were not accustomed to paying attention to precurrent skills like sitting still, concentration, articulation, or fine motor skills. These results coincide thoroughly with results of other interventions carried out with the same instruments, strategies, and procedures in other settings (Galindo, 2015).

On the other hand, the possibility should be considered that the children's improvements were not the result of the applied training, but of the work performed by teachers or due to the natural maturation process. It is still necessary to demonstrate that the positive behaviors were the direct result of the applied training programs.

Methodologically, these training programs represent a problem because it is difficult to compare a collection of such different cases since each child had a special problem and the training program was tailored for them.

In the next study, a quasi-experimental multiple baseline design (Johnston and Pennypacker, 2009) was applied in an attempt to resolve this issue. In a former work (Galindo, 2015), the successive application of the variable training to four children seemed to confirm that the results were caused by the intervention programs. In the following study, a replication of that work is carried out.

## Training children with school failure problems in alentejo: study 2

### Materials and methods

#### Participants and setting

Three 10- to 11-year-old fourth graders were referred to the program because of academic problems. They had a similar SES, as they belonged to the lower middle class of a Portuguese village. The causes of their problems were not determined, but at least one had family problems (separation of the parents). They were selected according to the following criteria: (1) they belonged to an average Portuguese family without a history of immigration; (2) they had failed at least one grade; (3) they had no reports of intellectual incapacity; (4) they had been referred by the teacher because of similar academic problems; and (5) they had taken part without success in other programs for children at educational risk.

Child A, a girl aged 10, had repeated the 3rd grade and was at risk of failing again. According to her teacher, she had difficulties understanding texts and verbal instructions (comprehension) and a lack of concentration skills and motivation. As a consequence, she had problems in math, environment and Portuguese (reading and writing).

Child B, a boy of 11, had repeated the 3rd grade. He had had delayed language development and behavioral (emotional) problems since his parents had divorced, and had been treated by a speech therapist and a psychologist. According to his teacher, he was disorganized, had difficulties concentrating on tasks, language problems, hyperactivity, and a lack of motivation. He had comprehension problems, poor spelling (orthography) and other writing problems.

Child C, a boy, aged 10, had suffered a health problem with secondary effects on his motor skills, as well as having moved house and school several times. He had repeated the last preschool year because of a lack of the necessary skills. According to his teacher, he presented oppositional behaviors, concentration problems and lack of motivation, and was consequently experiencing difficulties in all subjects. Regarding Portuguese reading and writing, he committed numerous and varied spelling errors.

The institution was a public elementary school situated in Arraiolos, a village in the Alentejo with retention rates higher than average in Portugal (Direção-Geral de Estatísticas da Educação e Ciência (DGEEC), 2014). It was a mixed class, where 18 children in the 2nd (7 students) and the 4th grades (11 students) were taught together. Obviously, having students from two different courses in the same class can influence the development of student learning. Most of the children had already repeated a grade. Training sessions were conducted at a table in a separate room of the school.

#### Instruments and materials

The main instrument relied on three behaviorally designed training programs to be applied in verbal comprehension (VC), reading (R), and spelling/orthography (O). The programs were designed to solve the specific problems of the three children according to a set of objectives set by the teacher. All programs had the structure explained in Study 1: (1) general objective, (2) specific objectives, (3) definition of precurrents, (4) pre-test, (5) training packaged based on instructions, modeling, rehearsal, and feedback, and (6) post-test. A first difference with respect to programs applied in Study 1 related to the content: all skills were defined according to the handbooks and materials of the 4th grade. A second difference was that the same program was applied to all three children.

The second instrument, a package of behaviorally designed check-lists aimed to assess the level of performance of the child in a determined area were also applied, following the same explained strategies.

#### Procedure

The assessment began once parents gave written informed consent and the children were in agreement. Each child was evaluated according to the previously described strategy. It was verified that all three children had roughly the same academic problems. After assessment, Behavioral Skills Training was applied to the three children by an ABA trained tutor for 3 h a week (2 sessions × 1 ½ h) during 21 weeks. The tutor took charge of all 3 children and worked with them from the beginning to the end of intervention. Procedures were applied following the same lines of Study 1. All information gathered was used to design training programs for the children, according to a set of objectives set by the teachers. The target skills, defined as specific objectives, were the goal of the intervention. The percentage of correct responses in a given program attained before (pre-test) and after training (post-test) were the main data.

A multiple baseline design across subjects was used to evaluate the effects of the training problems, much in the way as that used by authors treating similar problems (Wood et al., 1998; Levingston et al., 2009; Krohn et al., 2012). In the current study, the package training as independent variable was applied successively to all three children.

**Figure 1:**
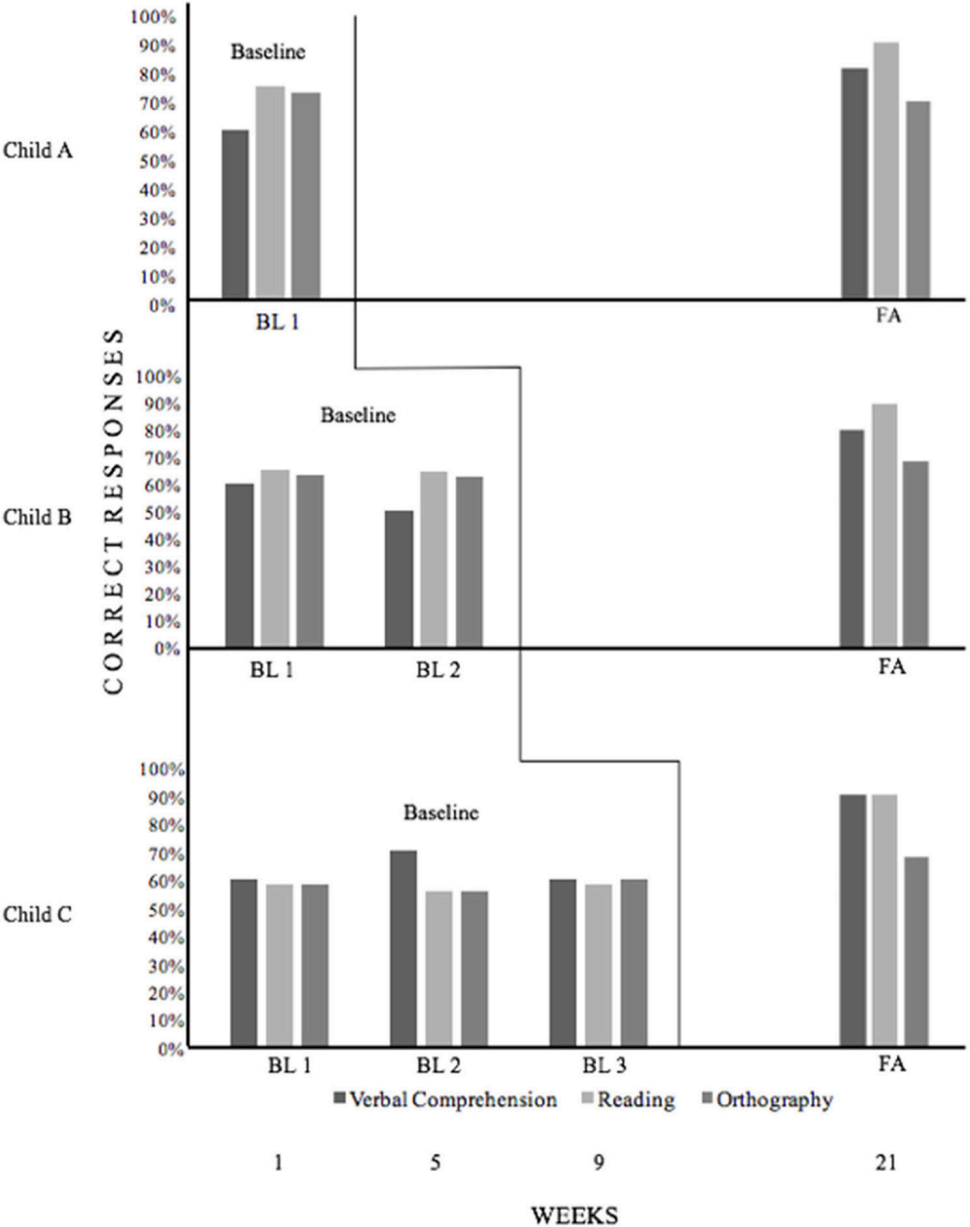
A multiple baseline analysis of results obtained in study 2 by three children. The figure shows in the ordinate the percentage of correct responses given by children A, B, and C across baseline (BL1, BL2, and BL3) and after training (FA), and in the abscissa the successive weeks in which an assessment was carried out. Children were trained in three skills (Verbal Comprehension, Reading, and Orthography); the onset of training was different for every child (according to Coradinho, 2015).

A baseline assessment (BL) based on the application of the training programs pre-test for VC, R, and O was conducted in week 1 (BL1), week 5 (BL2), and week 9 (BL3). The final post-test assessment (FA) was conducted in week 21. Following baseline assessment, all children received training consisting of multiple baseline. Training was then applied to the three children (see Figure 1). They were assigned randomly to one of 3 different training conditions:

CONDITION A: Child A was evaluated once before training (BL1). She then received 19 weeks of training and a final assessment (FA) was carried out in week 21.

CONDITION B: Child B was evaluated twice without training in weeks 1 (BL1) and 5 (BL2), and he was then trained for 15 weeks and a final assessment (FA) was performed in week 21.

CONDITION C: Child C was evaluated three times without training in weeks 1 (BL1), 5 (BL2), and 9 (BL3). He then received training for 11 weeks and a final assessment (FA) was made on week 21.

### Results

The results of the multiple baseline analysis for all three children participating in Study 2 are presented in Figure 1. Children A, B and C were trained in 3 subjects (verbal comprehension, reading, and spelling/orthography), but the onset of training was different for every child. Assessments were carried out in weeks 1, 5, 9, and 21. The percentage of correct responses attained by each child in every assessment is shown. Child A was evaluated once before training in week 1 (BL1) and once after 19 weeks training (FA). Child B was evaluated twice without training in weeks 1 (BL1) and 5 (BL2) and once after 15 weeks training (FA). Child C was evaluated three times without training in week 1 (BL1), week 5 (BL2), and week 9 (BL3) and once after 11 weeks' training (FA).

Overall, changes in all 3 children after training were evident. The most obvious took place in reading, where all three children attained 100% of correct responses in FA (week 21). An analysis of the individual results reveals other positive changes.

Child A showed clear increases from BL1 (week 1) to FA (week 21), advancing from 60 to 90% of correct responses in verbal comprehension, from 75 to 100% in reading, and from 73 to 89% in spelling (orthography). This improved performance seemed to have occurred in 19 weeks of training, i.e., 57 h.

Child B was evaluated without training in week 1 (BL1) and week 5 (BL2). Starting in week 6, a training procedure was applied for 15 weeks. The final evaluation (FA) was carried out in week 21. A comparison between BL 1 (week 1) and BL2 (week 5) showed little change. Indeed, change was negative, with the percentage of correct responses decreasing from 60 to 50% in verbal comprehension, from 65 to 64% in reading and from 63 to 62% in spelling (orthography). In other words, the child seemed to have gotten worse during training. On the contrary, a comparison of last BL2 (week 5) with FA (week 21) showed great increases, from 50 to 90% in verbal comprehension, from 64 to 100% in reading, and from 62 to 77% in spelling. This child's improvement seems to have occurred in a period of 15 weeks, i.e., 45 h.

Child C was evaluated without training in week 1 (BL1), week 5 (BL2), and week 9 (BL3); he was then trained for 11 weeks. A last evaluation (FA) was performed after training, in week 21. A comparison of BL1 (week 1) with BL2 (week 5) shows an increase of correct responses in verbal comprehension (from 60 to 70%), but decreases in reading and spelling/orthography (from 58 to 56% in both cases). A comparison of BL2 (week 5) with BL3 (week 9) shows a decrease from 70 to 60% in verbal comprehension, and slight increases in reading (from 56 to 58%) and spelling (from 56 to 60%). A comparison of BL1 (week 1) with BL3 (week 5) revealed that the student obtained the same values for verbal comprehension (60%) and reading (58%) and advanced slightly in spelling (from 58 to 60%). Finally, a comparison of BL3 (week 9) with FA (week 21) showed considerable increases in verbal comprehension (from 60 to 100%) and in reading (from 60 to 100%) and a modest increase in spelling (from 60 to 75%). This child's improved performance seems to have occurred with 11 weeks' training, i.e., 33 h.

### Discussion

These data seem to show that increases in positive responses occur consistently after training. Improvements without training were rare, but they did occur, such as, with child B (from week 1 to 5 in verbal comprehension), showing that positive changes can also result from other variables (probably the teaching at school). Nevertheless, decreases of correct responses occurred frequently without training, for instance with child B from BL1 to BL2 in all programs, revealing that regular teaching at school does not seem to improve these children's results and that a lack of systematic training might produce negative changes. The results of this study suggest that there was little change in children's performance following normal school activities (without training). When changes did occur, they were small and performance decreases were more common than increases. On the other hand, increases in academic and pre-academic behavior following the Behavioral Skills Training were evident in all cases and in all subjects. The following introduction of treatment resulted in considerable increases over baseline levels. These changes were obtained without corresponding changes in untreated baselines. The results of this multiple baseline analysis indicate that training generated improvement in all three children. It effectively enhanced performance in verbal comprehension, reading and spelling. All three children showed an obvious increase in the percentage of correct responses following the application of the training programs. This is to say that the multiple baseline analysis indicated that changes from baseline levels occurred only when training was directed to a specific child. The extent of the changes in the targeted child provides strong support for the effectiveness of the training package used. These results coincide with data obtained by Galindo (2015) with a similar quasi-experimental design, applying the same teaching strategies to students of a different environment, namely, children of a very low SES living in the slums of a big city, with problems of marginalization due to poverty or ethnic origins. In the Alentejo, these children were not living in conditions of exclusion. Their school failure problems coincided with family difficulties (Child B) or with health problems (Child C) or had no identifiable origin (Child A). In both multiple baseline studies, comparable results occurred across students, demonstrating the replication of the effects of the training programs.

## General discussion and conclusions

The results from both of the current studies show an apparent improvement of all children following Behavioral Skills Training. It seems that the observed advanced resulted from the intervention programs and not daily classroom teaching. These results confirm earlier findings (Galindo, 2008, 2013, 2015) and coincide with those of authors working in the same line in different countries (Carroll et al., 2003; DiLalla et al., 2004; Leppänen et al., 2004; Connor et al., 2005; Guevara and Macotela, 2006), especially in relation to the kinds of behavioral repertoires that should be addressed in the case of school failure, i.e., not only academic, but also precurrent behaviors.

“Precurrent behaviors are those that increase the effectiveness of a subsequent (current) behavior in obtaining a reinforce.” (Polson and Parsons, 1994, as cited by Levingston et al., 2009, p. 361). These behaviors are skills that are essential, as they form the basis for learning other skills. Many children fail to learn reading and writing for one of the following reasons: because they cannot discriminate forms and colors; or they lack motor coordination; or they have problems in verbal behavior. A lack of just one precurrent skill can have devastating consequences in the acquisition of reading, writing, or arithmetic. That is also the conclusion of Levingston et al. (2009) and Neef et al. (2003) in the teaching of arithmetic. Academic behavior is the main concern of teachers and scientists interested in school failure. Now, it is essential to define precisely, for each case, what the teacher is expecting and what the child can actually do. Social skills must be an integral part of any intervention. Many children with school failure problems also have behavioral problems and it is difficult to say if one is a cause or a consequence of the other. In either case, in order to solve academic failure, you might have to work simultaneously with social and academic skills (Monjas et al., 2014).

A word about the motivational is necessary. Most children with these kinds of problems do not want to learn. It is necessary to create a motivational system. A token economy seems to be the best solution.

In general terms, teachers reported significant positive changes in the children's behavior at school. Nevertheless, some complained that no changes were evident in academic behavior. It must be recognized that the developmental aims of the child and the academic aims of the teacher do not necessarily coincide.

It is difficult to determine the exact causes of the problems addressed, but all children belonged to a low SES and many had bad family relationships (parents in conflict or in a process of separation) or former health problems, or all those combined. Finding possible causes in the past development or in the present situation is always possible, but most of the time it is impossible to identify clearly the cause of school failure in individual cases. It seems that the individual problems of a child are a result of a set of bad coincidences at an important stage of development: the child missed the chance to learn the expected thing at the right moment for some reason and is not able to learn it later for other reasons. It is important to develop effective training procedures, independently of the factors causing the problem. Our results seem to show that programs of this kind can be applied successfully at school in order to train these kinds of skills, whose absence causes academic difficulties. Nevertheless, the present study is only a first contribution toward resolving school failure, for the following reasons:

1. Solving some academic problems still does not mean that the child will be successful. Additional training in other areas might be necessary.
2. More research on the long-term effects of these training programs is needed.
3. It is necessary to work with other problems at different school years.
4. It is necessary to work not only on the cognitive, but also the emotional problems of children with school failure.
5. A long-term observation of trained children is necessary.
6. It is necessary to develop more economic procedures to train children.

Although in this study the number of participants was relatively small, these ABA techniques already have a long history of success in training of people with intellectual, sensorial, physical, and social incapacities. Thus, it is not surprising to find they can be adapted to train children at educational risk. The current studies have demonstrated that BST techniques can be successfully applied to train children with problems of school failure. It has also been shown that a multiple baseline design can be a good choice for demonstrating the effects of training on a varied set of students. A further development of these training programs might constitute a sound psychological contribution to solving school failure as a social problem.

## Ethics statement

This research was carried on in the frame of an agreement between University of Evora (signed by Vice-Reitor Paulo Quaresma) and Malagueira School Group (signed by Diretora Isabel Gomes) and approved by an Ethic Commission of the University of Evora (President Prof. Armando Raimundo). Written informed consent was obtained from the parents of the participants.

## Author contributions

EG and AC: Conceptualization; EG: Methodology; MS: Formal analysis and supervision; HP and LG: Investigation; AC: Writing-review and editing.

### Conflict of interest statement

The authors declare that the research was conducted in the absence of any commercial or financial relationships that could be construed as a potential conflict of interest.
